# Regenerative Perspective in Modern Dentistry

**DOI:** 10.3390/dj4020010

**Published:** 2016-04-25

**Authors:** Mihnea Ioan Nicolescu

**Affiliations:** 1 Carol Davila University of Medicine and Pharmacy, Faculty of Dental Medicine, Histology and Cytology Division, Bucharest, 8 Eroilor Sanitari Blvd., RO-050474, Romania; mihnea.nicolescu@umf.ro; Tel.: +40-722-767-260; 2 Victor Babeș National Institute of Pathology, Radiobiology Laboratory, Bucharest, Romania

**Keywords:** regenerative dentistry, stem cells, translational medicine

## Abstract

This review aims to trace the contour lines of regenerative dentistry, to offer an introductory overview on this emerging field to both dental students and practitioners. The crystallized depiction of the concept is a translational approach, connecting dental academics to scientific research and clinical utility. Therefore, this review begins by presenting the general features of regenerative medicine, and then gradually introduces the specific aspects of major dental subdomains, highlighting the progress achieved during the last years by scientific research and, in some cases, which has already been translated into clinical results. The distinct characteristics of stem cells and their microenvironment, together with their diversity in the oral cavity, are put into the context of research and clinical use. Examples of regenerative studies regarding endodontic and periodontal compartments, as well as hard (alveolar bone) and soft (salivary glands) related tissues, are presented to make the reader further acquainted with the topic. Instead of providing a conclusion, we will emphasize the importance for all dental community members, from young students to experienced dentists, of an early awareness rising regarding biomedical research progress in general and regenerative dentistry in particular.

## 1. Introduction

Organs come from tissues; tissues are made up of cells. Each cell type has a certain lifespan, a time to carry on its specifically designated functions, after which it has to be replaced. The continuous renewal of the cellular pool takes place at different, specific paces for each tissue, and the key players in this are a peculiar cell category—the stem cells. These are special cells that possess a certain set of characteristics, and that are capable of renewing, to a limited extent, one or several differentiated cell types. Their number is considerably lower than that of other cellular types in the organ in which they reside, and their location is inside specific compartments, with very stable microenvironments, called stem cell niches. This is the physiological status quo. However, in particular cases when the need for the replacement rate or quantity is higher, local recovery mechanisms simply cannot cope anymore (e.g., defects larger than a critical size, as a result of injuries or various pathological conditions). This is the point where regenerative medicine intends to and, to some extent, may be able to help. *Regenerative* comes from “re-generate”, *i.e.*, “generate again” new cells, new tissues, and ultimately, new organs. The final goal is to re-establish the initial form and function of the damaged structure. In order to achieve this, one would either need to enhance the local healing processes or to replicate them using external materials; in other words, “to replace or regenerate human cells, tissues or organs; to restore or establish normal function” [1]. This necessity led to the development of a completely new translational science—*regenerative medicine*, and *regenerative dentistry*, subsequently. Its unique features allow its own scientific growth and regeneration based on bidirectional links with medical research, as well as with clinical therapeutic strategies, and last, but not least, with medical education. The major players of the regeneration process are represented by stem cells and their niches, which may be regarded together as a functional whole—the “stem cell unit” [2].

## 2. Stem Cell Units: Stem Cells and Their Niches

### 2.1. Stem Cells

The definition of stem cells (SC) is being continuously amended to accurately reflect the latest scientific findings. The scientist who coined the term “stem cells” was the 19th century German biologist Ernst Haeckel [3,4]. In principle, SC are cells capable of self-renewing and differentiation into one or several lineages of terminal specialized cells. However, there are different criteria that describe SC features. Among these, probably the most commonly used are potency and origin. Potency, often referred to as stemness, represents the cell’s capability to differentiate into one, several, many, or all tissue types (uni-, multi-, pluri- and totipotent cells). Totipotency, initially regarded as a zygote-only feature, has been recently redefined [5] as the ability of a single cell, as well as of its descendants, to colonize all the principal three lineages (*i.e.*, ecto-, mese- and endo-). Pluripotent SC are cells that are able to form any cell of the three germ layers, but not the extra embryonic tissues. Canonical embryonic stem cells (ESC) derive from the blastocyst inner cell mass, and possess pluripotent capabilities. It has been reported that a group of early blastocyst cells are able to retain their totipotency until a later stage [6]. To assess their differentiation potential, ESC are currently evaluated both *in vitro* and *in vivo*. *In vitro*, they either form embryoid bodies [7], or they differentiate into specific cell types (under particular conditions). *In vivo*, on the other hand, ESC form teratomas after subcutaneous injection into immunocompromised mice [8]. A distinct category is represented by the induced pluripotent stem cells (iPS). These are somatic cells that have been reprogrammed *in vitro*, manipulating transcription factors known to be highly expressed in ESC (Oct 3/4, Sox2, c-Myc and Klf4), also known as Yamanaka-factors, named after the Nobel Laureate scientist who published the procedure [9]. Recently, wider potential was attributed to *in vivo*–reprogrammed iPS cells, reported to achieve “a more plastic state than ESC”, and hence being capable of generating structures that also express extraembryonic markers [10]. With regards to the SC origin, one may group them into embryonic and adult stem cells. The latter are a particular category, which reside in their special aforementioned niches, capable of generating up to several types of progenitor, differentiated, and daughter cells (uni- and multipotent, respectively). Adult SC and their niche microenvironment are of high importance in regenerative medicine, since they are responsible for *in vivo* tissue homeostasis and its repair/regeneration.

### 2.2. Stem Cell Niches

Stem cell properties may be regarded as a result of their molecular repertoire, but in fact they are strongly influenced and regulated by their immediate external microenvironment, collectively named the niche [11,12,13]. Schofield was the first to hypothesize that other cells might influence the behavior of stem cells inside a “niche” [14]. Chemical interactions are not the only input that SC are receiving. Spatial positioning inside the niche has also proved the “significance of the niche for stem-cell fate determination” [15]. Thus, SC may remain quiescent or actively engaged in contributing to local tissue homeostasis maintenance, depending on the actual three-dimensional location in relation with highly specialized niche compartments. Between SC and terminal progenitors there is a population of intermediate committed progenitors, known as transit amplifying cells, which might regain their stemness under certain conditions [16]. Notably, the niche concept is a universal one. It has been pointed out in plants [17], worms (*Caenorhabditis elegans*) [18] and insects (*Drosophila*) [19]. However, each particular niche seems to be unique, even in the same organism, at least in what concerns cellular composition. A common feature of niches is the physical proximity to SC, and, not unexpectedly, their capacity of stimulating SC quiescence and survival, and of maintaining their particular undifferentiated state [2]. Its components include the extracellular matrix, vascular [20] and neural supply [21], stromal cells [22,23] and, of course, SC and their progenies [24] in various differentiation stages. The continuous feedback sent and received among the intrinsic components, as well as systemic connections via nerves, blood vessels and possible intermediate cells, such as telocytes [25,26], contribute in maintaining the proper niche function in an inductive/restrictive microenvironment.

### 2.3. Stem Cells Isolated from Oral Cavity

It has been a tendency during the last decades to label each new adult SC in various organs as a “mesenchymal stem cell”, or “stromal stem cell”. Dominici defines the most accurate characterization of their features [27], naming them “multipotent mesenchymal stromal cells” (MSC). Briefly, the conditions stated by Dominici *et al.* [27] for a cell population to fall under the MSC category are:

- to adhere to plastic in standard culture conditions;
- to express CD105, CD73 and CD90 markers (and also to be negative for CD45, CD34, CD14/CD11b, CD79α/CD19, HLA-DR); and
- to be able to differentiate *in vitro* into mesenchymal lineages: osteoblasts, chondrocytes and adipocytes.

Shi’s group identified a MSC type inside the dental pulp in the early 2000s. These cells, named dental pulp stem cells (DPSC), are able to produce fragments similar to dentine in immunocompromised mice [28,29]. The SC features of DPSC (self-renewal and lineage development) have been assessed by Vasanthan *et al.* in a study of the expressed MicroRNAs, in which they concluded that several small regulatory non-coding RNAs are acting together to support the functional switch between stemness and further differentiation [30]. In addition to DPSC, different SC populations harbored by the teeth and related structures have been isolated and characterized: PDLSC (periodontal ligament stem cells, [31]), SCAP (stem cells from the apical papilla [32,33]), SHED (stem cells from human exfoliated deciduous teeth, [34,35]), or DFSC (dental follicle stem cells [36,37]). Various groups compared different oral SC populations, finding advantages and disadvantages for each category, and hence keeping up the momentum of the dental SC research. Thereby, Shoi reported the higher proliferation capacity of DFSC compared to DPSC [38], and Lee stated that SHED is a better source of multipotent SC than the DPSC of supernumerary teeth [39]. Dentition was not the only source of SC inside the oral cavity. A plethora of cell populations derived from soft oral tissues exhibited SC features *in vitro*: OMNEC (oral mucosal non-epithelial cells, [40]), OMLP-PC (oral mucosal lamina propria progenitor cells, [41]), OMSC (oral mucosa stem cells, [42]). As might be expected, gingiva is also an important source for different SC types. There have been reports on GMSC (gingival-derived mesenchymal stem cells, [43,44,45]), GTMSC (gingival tissue derived stem cells, [46]) or GMPC (gingival multipotent progenitor cells [47]). 

As an iPS alternative to oral adult MSC, oral fibroblasts have been reprogrammed into TiPS cells (*tentative* iPS) [48]. Reciprocally, dental cell populations served as a source for reprogramming into iPS cells by using cocktails of different iPS factors (for a detailed review, see p. 301 in [49]). Another major feature of MSC is represented by their immunomodulatory capabilities [50]. Zhang has reported anti-inflammatory properties of GMSC, mainly mediated by IL-10, making them capable of ameliorating experimentally-induced colitis [45]. Several reviews have compared immunological properties of MSC of different origin (either oral, bone marrow or adipose) and their putative clinical potential [51,52], concluding that oral MSC should be taken into account as a viable (and, in certain conditions, more immunoactive) alternative to the less-accessible classical bone marrow MSC. On the other hand, it has been shown that paracrine secretion (e.g., microvesicles, exosomes) is an important mechanism used by SC in repairing damaged tissues [53]. This opens the way for a new class of non-cellular therapy, based mostly on SC-derived microvesicles, with very promising preliminary results [54,55]. Oral MSC represent a convenient source of autologous SC, especially due to the high accessibility of oral tissues, but also for their specific immunomodulatory features [56]. Dental-derived MSC possess remarkable tissue-regenerative properties, with extensive potential clinical applications. The most explored use for adult oral MSC has been, naturally, in dental research. Thus, niches for dental therapy applications were quickly addressed in almost all aspects of clinical dentistry—endodontics, periodontics, orthodontics and the list goes on.

It is noteworthy that the attractive concept of *de novo* creation of a whole tooth has also gained increased attention in the last years. The reciprocal stimulation occurring between epithelial and mesenchymal cells during tooth development and how this process might be replicated and translated into creating a whole bio-engineered tooth has been extensively studied [57,58,59].

## 3. Regenerative Endodontics

Early regenerative endodontics (RE) stems from the middle of the 20th century, when Hermann used calcium hydroxide in a vital pulp amputation case [60,61]. According to the same authors, RE consists of the research of SC, growth factors, tissue culture and materials in tissue engineering [61]. It is not the purpose of this review to exhaustively deal with all the regenerative techniques in dental treatment, but to give notice of their existence to students and dental professionals. Any discussion of RE should start by referring to the pulp-dentine complex, comprised of pulp core, odontoblasts, predentin and dentin, and to point out that they should be regarded as a whole unit, due to their particular interdependent relation. There have been reported variances of composition in dentine types related to age, following changes in odondoblasts transcriptome [62]. Since the age of odontoblasts is reflected in their dentin production an ideal endodontic treatment, especially in the case of immature teeth with necrosis of the pulp, should aim to regenerate a functional pulp tissue [63]. One of the classical treatments is apexification, which is still under debate regarding the use of calcium hydroxide *vs.* mineral trioxide aggregate [64,65,66]. A step further on the RE path is represented by the revascularization procedure [67], in which a provoked apical bleeding leads to the formation of a blood clot, with the intention of further using it as a scaffold, for local stem cells to repopulate the pulp-dentine territory. It is becoming increasingly clear that a better result might be best yielded by a combination of the right cells, scaffolds and signals. An up-to-date review of the tissue engineering studies in RE has been recently published [49]. One of the latest approaches in RE treatment is the combined use of DPSC and a silk fibroin scaffold with b-FGF [68]. Scaffolds containing growth factors [69] are currently regarded as one of the most physiological alternatives for replacing tissue defects. A similar methodology has also been reported as a possible periodontal treatment [70].

## 4. Regeneration of the Alveolar Bone and Periodontal Tissues

Teeth and periodontal ligaments contribute to the total stress/strain equilibrium in the mandibular bone. The mandible behaves, mechanically speaking, as if the space occupied by teeth and their support apparatus was an empty one [71]. Therefore, when a structure, either implant alone or implant plus bone, is used to fill in the defect resulting after a dental loss, it modifies the total bone stiffness. Thus, the strain stimulus required in bone mass maintenance is no longer attained, resulting in a reduction of the alveolar ridge *ibidem*. Taking this into account, we may state that the desired outcome of a successful regenerative therapy is to achieve both a morphological as well as a functional replacement of the tissue(s). In other words, one has to keep in mind that simply replacing lost teeth by rigid components would eventually lead to a bone remodeling/resorption in the long term, decreasing the alveolar bone mass and consequently affecting the whole craniomaxillofacial functionality and equilibrium. From the point of view of the orthodontist or the maxillo-facial surgeon, there are situations that would mostly benefit from a higher alveolar bone offer, either in length, height, or both. One of the surgical procedures used for this purpose is bone distraction [72]. Injection of bone marrow MSC into the created osseous defect promotes a faster bone formation, thus leading to the shortening of the treatment duration [73]. The regenerative response in the case of craniofacial bone defects was shown to be enhanced by the local delivery of “tissue repair cells”, in fact a mixture of bone marrow–derived predominant CD90^+^ and CD14^+^ cells [74]. As mentioned earlier, SC are not the only players on the regenerative team. The focus of SC research has lately slightly shifted towards a close comprehension of the molecular details of cell-signaling cascades. The multiple cross-interactions between epithelial and mesenchymal counterparts during development are mediated by several signaling molecules [75], including proteins such as BMP (Bone Morphogenetic Protein), Wnt, FGF (Fibroblast Growth Factor) or Shh (Sonic Hedgehog). Gradually uncovering the mysteries of the complex molecular interactions between all the cellular and subcellular components inside the SC niche allows a progressive understanding of the physiological workflow that underlies first the efficient tissue formation during development, and secondly its subsequent maintenance, repair and regeneration in adulthood. An equally important role is played by the scaffold, which may currently be customized to match the periodontal defect and enhance local tissue regeneration. A favorable route of using growth factors as an adjuvant treatment has been proven in a study on beagle dogs, in which the combined delivery of PDGF-B and BMP7, using a mesoporous bioglass/silk fibrin scaffold, led to an increased recruitment of local SC surrounding the defect [70]. Nevertheless, some consider other sources apart from the oral cavity would yield a higher number of SC to be used in periodontal tissue engineering [76]. These sources include bone marrow [77], adipose tissue [78], even iPS [79] or ESC [80].

## 5. Regenerative Approach in Salivary Gland Dysfunction

Salivary glands’ (SG) loss of function leads to a chronic lack of saliva, with repercussions in digestion, mastication, phonation, and ultimately in quality of life. The multimodal treatment of many head and neck cancers frequently includes local irradiation, affecting the SG, which are highly radiosensitive [81]. One must not neglect, though, that xerostomia (dry mouth) might also be caused by primary afflictions, such as Sjögren’s Syndrome, an autoimmune pathology that affects one in 1400 Europeans [82], or by side effects of certain medications. A classical model in SG research is the “ligation” animal model. A small metal clamp is attached on the excreting duct of a salivary gland, and the ‘ligated’ gland undergoes atrophy, but starts to regenerate once the clip is removed. The regeneration would, however, take place only if the neural supply of the gland is not injured [83], thus proving the importance of the crosstalk between different components of the adult SC niche in SG and the intrinsic SG recovery potential. A more recent method, utilized in SG regeneration research, is the culture of salivary spheres, or salispheres [84]. Some of the cells harvested from salivary glands, cultured in a favorable environment, tend to group together in small multi-cellular aggregates, called salispheres [84]. Salivary glands of irradiated mice, lacking the spontaneous recovery exhibited by the deligated SG mentioned above, have shown remarkable regeneration when injected with selected c-Kit^+^ cells from salispheres [85]. 

Translating the knowledge on the dual embryological origin of SG (epithelial and mesenchymal) into experimental SG tissue engineering has been attempted in several ways. The use of DPSC as an inductive mesenchyme was successfully used in a murine model [86]. Functional regeneration of SG has also been reported by transplantation of a bioengineered SG germ [87]. 

## 6. Is There a Niche for Regenerative Dentistry in the Dental Curriculum?

All the above-mentioned examples illustrate how the progress achieved in genetics, molecular biology, stem cell research, biomaterials and tissue engineering transformed regenerative dentistry from utopia to something tangible. Regenerative dentistry became a field on its own, with a real-time echo in clinical practice. It is capable of liaising medical sciences from early phases of undergraduate dental education with patient clinical care, an attribute of the concluding training phases of the curriculum. Therefore, it is of utmost importance for dental students to be aware and stay informed about the existence and progress of regenerative dentistry, especially for widening their view on the developmental and repair processes that take place in different areas of the oral and maxillofacial territory. This would offer them the possibility of a real informed choice on how to handle various clinical situations, laying the grounds for a better understanding of the momentousness of conducting a synergistic treatment, coordinating with and enhancing the body’s own mechanisms of repair and regeneration. Secondly, every dental practitioner should know the clinical theraputic potential of the regenerative approach in almost every aspect of the pathology that they may encounter, including in endodontics, periodontology, orthodontics, oral pathology or salivary glands diseases.

In terms of practicality, there are several ways in which an education provider may choose to deliver the information to trainees. As Huttly noted, “Students’ learning and experiences are the most important aspects of a successful curriculum” [88]. The outcome is in direct relation to the degree in which the audience is willing to be an active participant, as opposed to just a passive information recipient. One of the successes of good evidence-based medical teaching is to focus on the learners’ actual learning needs [89]. The attention of dental care professionals needs to be captured by presenting many data and case reports relevant to their specific clinical niche. The key element is to make the clinicians understand how regenerative dentistry will help their patients to achieve an improved healing and yield better morphological and functional outcome, which will ultimately lead to an increase in their contentment and gratitude.

After all, it was Voltaire who said: “The art of medicine consists in amusing the patient while nature cures the disease”. Most probably, the future of regenerative medicine will allow physicians to recreate the conditions of a proper *in situ* microenvironment, so that endogenous healing will prevail over external maneuvres, for a swift and durable patient recovery.

## Abbreviations

The following abbreviations are used in this manuscript:

SC: stem cells
ESC: embryonic stem cells
iPS: induced pluripotent stem cells
MSC: multipotent mesenchymal stromal cells
DPSC: dental pulp stem cells
RNA: ribonucleic acid
PDLSC: periodontal ligament stem cells
SCAP: apical papilla stem cells
SHED: stem cells from human exfoliated deciduous teeth
DFSC: dental follicle stem cells
OMNEC: oral mucosal non-epithelial cells
OMLP-PC: oral mucosal lamina propria progenitor cells
OMSC: oral mucosa stem cells
GMSC: gingival-derived mesenchymal stem cells
GTMSC: gingival tissue derived stem cells
GMPC: gingival multipotent progenitor cells
TiPS: tentative induced pluripotent stem cells
RE: regenerative endodontics
b-FGF: basic fibroblast growth factor
SG: salivary glands
BMP: bone morphogenetic protein
FGF: fibroblast growth factor
Shh: sonic hedgehog
PDGF: platelet-derived growth factor
